# Phylogenetic analysis and clinical characteristics of the co-occurring mutations in HA and NA genes of influenza A(H1N1)pdm09 viruses during 2015–2017 in Beijing, China

**DOI:** 10.1186/s12985-020-01446-3

**Published:** 2020-11-19

**Authors:** Yafen Liu, Yue Wang, Baiyi Liu, Xu Cong, Ying Ji, Xiaolin Guo, Yan Gao

**Affiliations:** 1 Department of Infectious Diseases, Peking University Hepatology Institute, Peking University People’s Hospital, No. 11, Xizhimen South Street, Xicheng District, Beijing, 100044 People’s Republic of China; 2 Peking University Hepatology Institute, Peking University People’s Hospital, No. 11, Xizhimen South Street, Xicheng District, Beijing, 100044 People’s Republic of China

**Keywords:** A(H1N1)pdm09, Epidemiology, Evolution, Variation, Co-occurring mutations

## Abstract

**Background:**

Influenza A(H1N1)pdm09 viruses have undergone rapid evolution, and in recent years the complementary and antagonistic effects of HA and NA have gathered more attentions; however, the effects of co-occurring mutations in HA and NA on the patients’ clinical characteristics are still poorly understood. In this study, we analyzed molecular epidemiology and evolution of A(H1N1) pdm09, explored co-occurring mutations of HA and NA, and investigated effect of co-occurring mutations on patients’ clinical features.

**Methods:**

A(H1N1)pdm09 was confirmed by reverse transcription-polymerase chain reaction. HA and NA genes were sequenced and phylogenetically analyzed. Clinical characteristics of the co-occurring mutations were analyzed statistically.

**Results:**

By analyzing the HA and NA gene sequences of 33 A(H1N1)pdm09 viruses during the 2015–2017 influenza season, we found that all the viruses shared high similarities to each other and the HA genes of these viruses exclusively belonged to subclade 6B.1A. Several unreported substitutions in HA and NA proteins were observed, furthermore, co-occurring mutations of HA-V169T, A278S, E508G, D518E and NA-V67I were detected in 30.3% (10/33) A(H1N1)pdm09 virus strains when comparing with vaccine strains A/California/07/2009 and A/Michigan/45/2015 (H1N1). Sore throat was significantly associated with co-occurring mutations in HA and NA of A(H1N1)pdm09 (χ^2^, *P* < 0.05).

**Conclusions:**

Co-occurring mutations in HA and NA were detected in A(H1N1)pdm09 isolated during 2015–2017 in Beijing. Symptomatically, sore throat was associated with co-occurring mutations in HA and NA of A(H1N1)pdm09. Therefore, studying the effect and mechanism of co-occurring mutations in HA and NA on patients’ clinical features is of note needed.

## Background

Influenza is one of the most important respiratory infections of humans with significant morbidity and mortality each year worldwide. Of the four types of influenza virus, type A is the most virulent, and occasionally causes large-scale global pandemics [1]. Iuliano et al*.* estimated that 291,243–645,832 seasonal influenza-associated respiratory deaths occur annually worldwide [2, 3]. Under selective pressure from the host immune system, antigenic epitopes of influenza virus hemagglutinin (HA) have continually evolved, termed antigenic drift, and influenza viruses evolve by genetic reassortments, leading in some occasions to life threatening pandemics [4]. The HA gene mutates the fastest of the eight genes in influenza A viruses, followed by the neuraminidase (NA) gene [5]. Evolutionary analysis of previous studies showed that influenza A(H1N1) pdm09 virus was derived from several viruses circulating in swine, and that the initial transmission to humans occurred several months before recognition of the outbreak [6]. Therefore, active epidemiological monitoring of influenza virus at molecular level is necessary [7], which is conducive to understand the evolution of influenza virus, in order to better select vaccine strains for effective prevention [8].

New variants of HA and NA might contribute to the emergence of new clinical characteristics, for example, HA-D239E mutation was associated with mild infection, been less severe than HA-D239G and D239N [9], and NA-H275Y, NA-E119D conferred resistance to neuraminidase inhibitors [10, 11]. However, the interactions between virus evolution, epidemiology and human behavior were complicated [12]. HA and NA are the two major surface glycoproteins of influenza A viruses, both of which recognize the same molecule (sialic acid, SA) with conflicting activities [13]. In recent years, more attentions have been paid to study the effects between HA and NA [14, 15]. By recognizing and binding to SA, the HA allows virus attachment and entry into host cells, and the NA ensures the release of progeny virus [16]. A balance of competent HA and NA activities have been reported to be critical for efficient influenza virus replication and human-to-human transmission [17]. Since there are complementary and antagonistic effects between HA and NA, whether the co-occurring mutations (HA and NA mutated simultaneously) of A(H1N1)pdm09 have any impact on patients’ clinical characteristics is still unknown.

Since 2009, we have monitored and made relevant statistics on the incidence of influenza viruses in Beijing. Studies on the evolution of surface antigen site mutations have performed using the viruses circulated in 2012–2014 [18–20]. In this study, we continued to investigate the molecular evolution and amino acid variation characteristics of HA and NA of A(H1N1) pdm09 during the 2015–2017 influenza seasons in Beijing, China. Meanwhile, we intended to explore co-occurring mutations of HA and NA in the new sites and analyzed effect of co-occurring mutations on patients’ clinical features.

## Materials and methods

### Patients and sample collections

The study population included outpatients (≥ 16 years) who sought medical attention in the Department of Infectious Disease of Peking University People’s Hospital (PKUPH), a national influenza surveillance sentinel unit, at which at least 40,000 patients from all Beijing districts are seen annually. During the 2015–2017 influenza season (December to the following March), a total of 11,112 nasal swab specimens were obtained from influenza-like illness patients. The samples were screened for influenza A and B viruses by using colloidal gold method. The influenza A positive samples were immediately placed in virus transport media tubes and were stored at − 80 °C within 24 h.

### RNA extraction and influenza A subtyping

We extracted RNA from samples using the QIAamp Viral RNA Mini Kit (Cat. No.52904, Qiagen, Hilden, Germany) and performed the reverse transcriptase polymerase chain reaction (RT-PCR) with a commercial kit (Cat. No. 18080051, Invitrogen, Carlsbad, CA, USA), following the manufacturer’s instructions. The complementary DNAs (cDNAs) generated from the reverse transcription were stored at − 20 °C until use.

Subtypes H1 and H3 were identified by specific primers for influenza A positive samples. H1- and H3-specific primers were listed as follows: H1 forward primer (5′-ATGAAGGCAATACTAGTAG-3′ and 5′-GATTGCAATACAACTTGTC-3′), reverse primer (5′-GATCGGATGTATATTCTGAAATGG-3′ and 5′-AATACATATTCTACACTGTAGAGACCCA-3′); H3 forward primer (5′-AAAGCAGGGGATAATTCTA-3′ and 5′-GGTTACTTCAAAATAC-3′), reverse primer (5′-ATTGCTGCTTGAGTGCTT-3′ and 5′-AGTAGAAACAAGGGTGTTTT-3′); N1 forward primer (5′-AGCAAAAGCAGGAG-3′ and 5′-GACAGGCCTCATACAAGATCTTC-3′), reverse primer (5′-GTGATAATTAGGGGCATTC-3′ and 5′-AATTACTTGTCAATGG-3′); N2 forward primer (5′-AGCAAAAGCAGGAGT-3′ and 5′-GAACTTGTRCAGTRGTAATG -3′), reverse primer (5′-CGACATGCTGAGCACTYCCTGAC-3′ and 5′-AGTAGAAACAAGGAG-3′).

### Gene sequencing

Samples tested positive for seasonal H1 subtypes were then randomly selected for analysis. For sequencing the HA and NA genes, high-fidelity thermostable DNA polymerase (Cat. No.11304011, Invitrogen) was used with primers of pdmHA-F (5′-TGTAAAACGACGGCCAGTATGAAGGCAATACTAGTAG-3′) and pdmHA-R (5′-CAGGAAACAGCTATGACCAATACATATTCTACACTGTAGAGACCCA-3′) for HA genes or pdmNA-F (5′-TGTAAAACGACGGCCAGTAGCAAAAGCAGGAG-3′) and pdmNA-R (5′-CAGGAAACAGCTATGACCAATTACTTGTCAATGG-3′) for NA genes. Products were sequenced using the Sanger method. The PCR amplification system included the cDNA template (4 μl), Autoclaved, distilled water (12.1 μl), 10X High Fidelity PCR Buffer (2 μl), 50 mM MgSO_4_ (0.6 μl), 10 mM dNTP Mix (0.4 μl), 10 µM forward primer (0.4 μl), 10 µM reverse primer (0.4 μl), and Platinum® Taq DNA Polymerase High Fidelity (0.1 μl of 5 U/µL). The PCR conditions for HA genes of influenza A were: 94 °C for 3 min, followed by 35 cycles of 94 °C for 0.5 min, 55 °C for 0.5 min and 72 °C for 1.5 min, with extension at 72 °C for 7 min. The PCR conditions for NA genes of influenza A were: 94 °C for 3 min, followed by 40 cycles of 94 °C for 0.5 min, 52 °C for 0.5 min and 72 °C for 70 s, with extension at 72 °C for 7 min. PCR products were analyzed by the method of electrophoresis.

### Phylogenetic analysis of HA and NA genes

Nucleotide sequences of H1 or N1 influenza viruses which were relative to our studied human A(H1N1)pdm09 influenza strains were obtained from the National Center for Biotechnology Information (NCBI) database. All HA and NA gene sequences were aligned using ClustalW 1.83. Phylogenetic analysis of HA and NA gene sequences was performed using MEGA software version 7.0. Sequences of H1 and N1 genes in our study have been deposited into NCBI with the accession number MN636362-636389, MN636393-636406, and MN636408-636423.

### Statistical analysis

Statistical analysis was performed using SPSS statistical software version 22.0 (SPSS Inc., Chicago, IL, USA). Continuous variables were expressed as means ± SD or median (interquartile range) and discrete variables as counts (percentage). Two-group comparisons of normally distributed data were performed with the independent samples t-test. Frequency comparisons were made with the χ^2^ test. *P* values < 0.05 were considered statistically significant.

## Results

### Epidemics characteristics of influenza virus

**Fig. 1:**
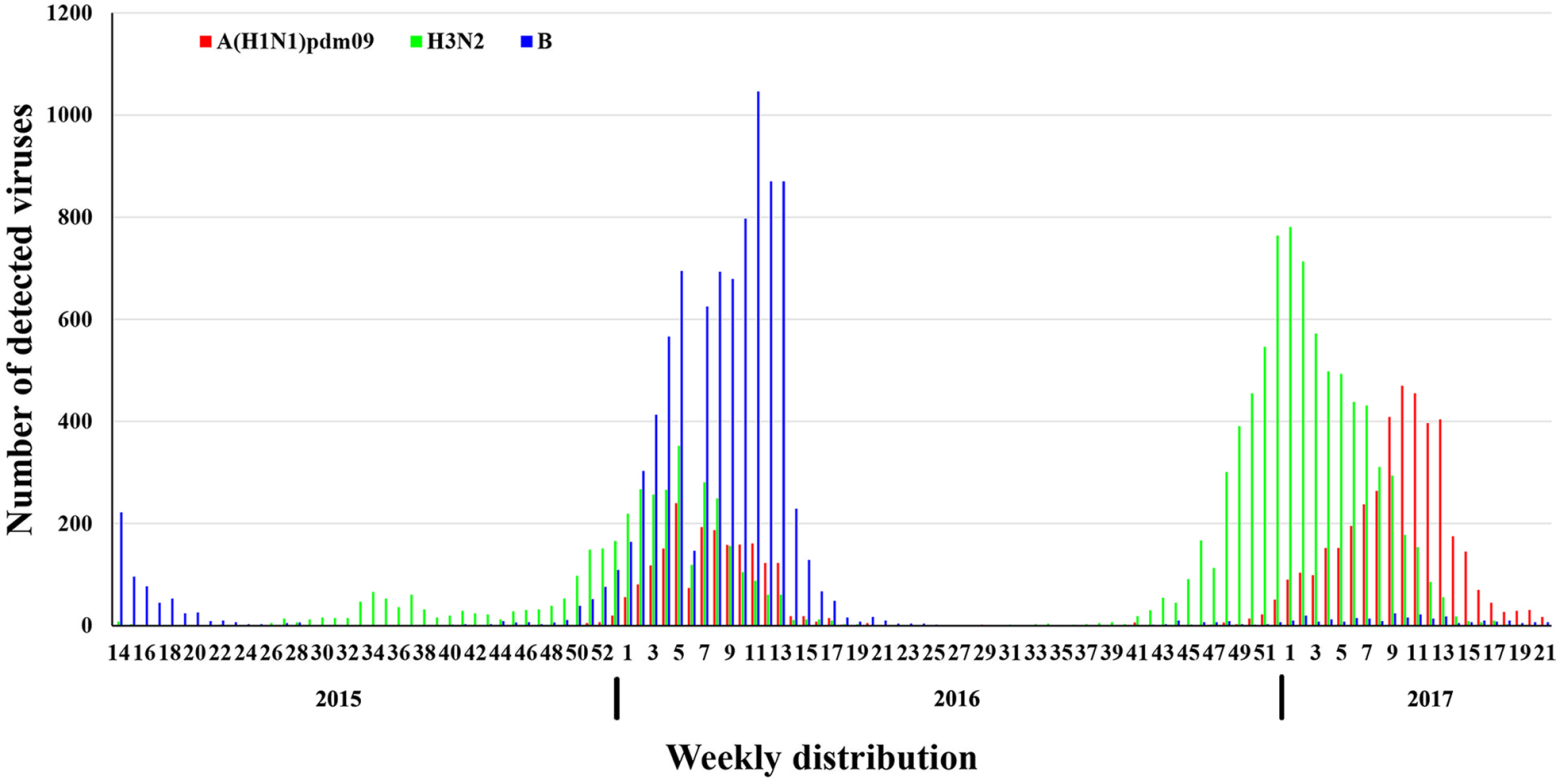
Distribution of influenza viruses in northern China. The data were from weekly data of Influenza Laboratory Surveillance Network during April 2015 to May 2017. Red is A(H1N1)pdm09, green is influenza H3N2, and blue is influenza B

The Chinese Center for Disease Control and Prevention (CDC) organized the Influenza Laboratory Surveillance Network for nationwide monitoring on patients with influenza-like illness in China. In order to elucidate the epidemiology of influenza virus in northern China during April 2015 to May 2017, we summarized the weekly data during the influenza epidemic. As shown in Fig. 1, influenza B viruses co-circulating with A(H1N1)09pdm and H3N2 viruses in 2015–2016 flu season in northern China. The prevalence of influenza B viruses has been maintained at a high-level during February and March in 2016. In contrast, Influenza A viruses were predominant during the 2016–2017 flu season. H3N2 epidemic started earlier and peaked at the beginning of 2017, then A(H1N1)pdm09 gradually increased, and peaked in the middle of March (Fig. 1).

### Influenza A subtyping

**Fig. 2:**
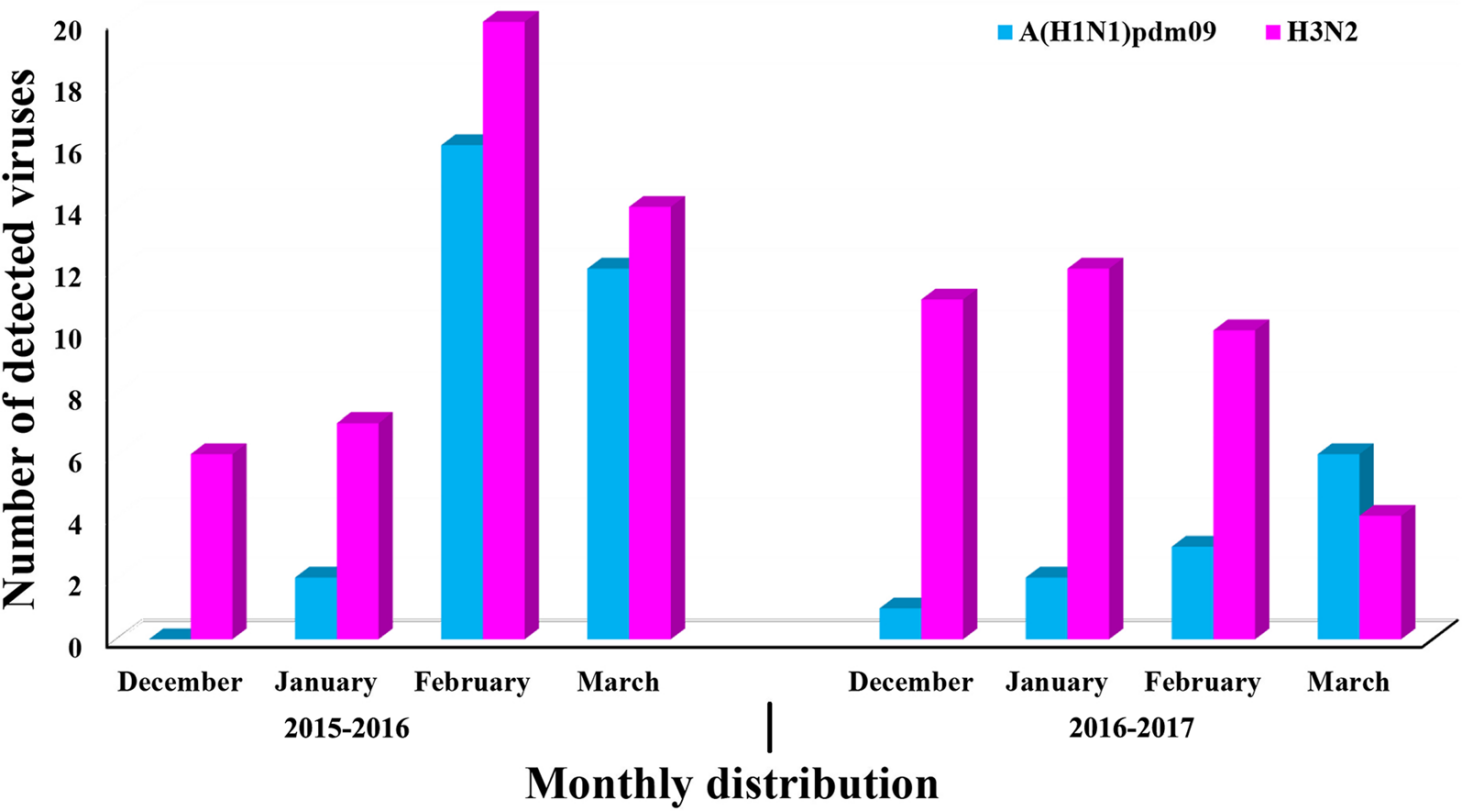
Monthly distribution of 126 nasal swab samples tested positive for influenza A virus

During the 2015–2017 influenza season, 126 nasal swab samples tested positive for influenza A virus using colloidal gold method were collected from the Department of Infectious Disease of PKUPH. A(H1N1)pdm09 were detected in 42 samples and the remaining 84 samples were H3N2 positive. As shown in Fig. 2, the prevalence situation of influenza A viruses in Beijing in this study was similar to those in Northern China. H3N2 viruses were co-circulating with A(H1N1)pdm09 viruses in 2015–2016. However, the H3N2 viruses were predominant during the 2015–2017 flu season (Fig. 2).

### Homology analysis of Hemagglutinin (HA) and Neuraminidase (NA) genes

The HA and NA genes of the 33 A(H1N1)pdm09 viruses were fully sequenced and phylogenetically characterized. As shown in Additional file 1–4: Table 1–4, HA genes of the viruses shared 96.9–100% nucleotide similarity and 97.9–100% amino acid identity to each other. NA genes of the viruses shared 97.3–100% nucleotide similarity and 96.3–100% amino acid identity to each other. In addition, A(H1N1)pdm09 isolates in this study shared 96.9–98.5% nucleotide similarity and 96.3–97.8% amino acid identity of their HA and NA genes to A/California/07/2009 (H1N1) vaccine strain, respectively. Moreover, A(H1N1)pdm09 isolates shared 97.5–100.0% nucleotide similarity and 97.8–100% amino acid identity of their HA and NA genes to A/Michigan/45/2015 (H1N1) vaccine strain, respectively. These results suggest that these viruses contained similar gene constellation and possessed high gene identity to the vaccine strain A/Michigan/45/2015 (H1N1).

### Phylogenetic analysis of HA and NA genes of A(H1N1)pdm09 viruses

**Fig. 3:**
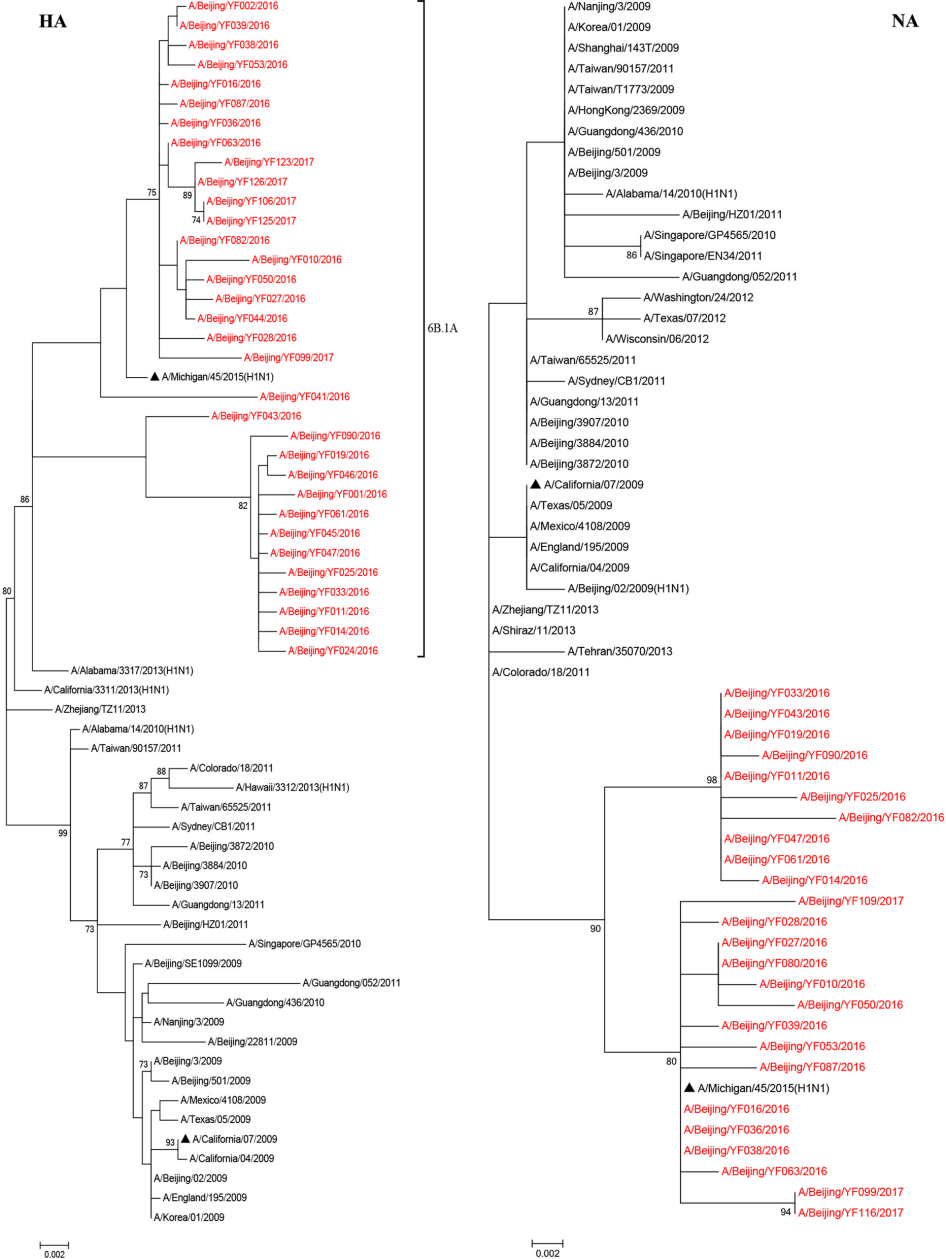
Phylogenetic tree based on HA and NA nucleotide sequences of A(H1N1)pdm09 from 2009 to 2017. Phylogenetic analysis of HA and NA gene sequences was performed with the Tamura 3-parameter model which was the best fit for our data using MEGA software version 7.0, with gamma-distributed rates. The reliability of the maximum-likelihood tree was run by bootstrap analysis with 1000 replications. ▲ Represents the vaccine strain

To determine the evolutionary relationship of 33 A(H1N1)pdm09 viruses detected during 2015–2017, HA and NA gene sequences were compared with other A(H1N1)pdm09 sequences on NCBI. Phylogenetic analyses of the HA genes showed although the tested A(H1N1)pdm09 viruses clustered into two subclades, they fell together with the vaccine strain A/Michigan/45/2015 (H1N1), and belonged to subclade 6B.1 (Fig. 3). Like HA genes, the NA genes showed the same evolutionary pattern. These results suggest that these viruses shared common evolutionary lineages to the vaccine strain A/Michigan/45/2015 (H1N1).

### Substitution analysis of HA and NA genes

Compared with the vaccine strains of A/California/07/2009 and A/Michigan/45/2015 (H1N1), 33 strains of A(H1N1)pdm09 virus in this study exhibited several unreported substitutions, as shown in Table 1. HA of all strains had S220T, K180Q, S202T mutations and NA V264I mutation. The oseltamivir resistance substitution of NA-H275Y has not been observed in these strains.

**Table 1:** Unreported substitutions of 33 strains of A(H1N1)pdm09 virus in this study

HA		NA	
Substitutions	n	Substitutions	n
T19P	4	T16I	1
R26I	1	G41D	1
S86T	1	N50Y	1
V169T	13	E57K	1
S181T	1	V67I	10
G254D	1	T72I	1
A278S	13	A86T	1
T327I	1	P93S	1
I341V	1	F115L	4
I435V	1	Y155H	1
E508G	12	P198S	1
D518E	13	T381I	3
		N449T	4
		S450V	4
		D451T	4
		T452L	4
		V453W	4

### Co-occurring mutation analysis in both HA and NA proteins

Based on above substitution analysis of HA and NA genes, co-occurring mutations of HA-V169T, A278S, E508G, D518E and NA-V67I were detected in 30.3% (10/33) A(H1N1)pdm09 virus strains when comparing with vaccine strains A/California/07/2009 and A/Michigan/45/2015 (H1N1). To investigate the associations with clinical characteristics and the co-occurring mutations in HA and NA, we collected medical data from these ten patients in which the above co-occurring mutations were detected, including demographic characteristics, symptoms, laboratory test results, and whether complicated by pneumonia (Table 2). Another ten patients with no co-occurring mutations detected were selected randomly as controls. By comparing these two groups, we found that sore throat was more common in co-occurring mutations [100.0% (10/10) vs. 50.0% (5/10), *P* < 0.05]. No statistically significant difference was detected for other clinical characteristics. This result indicated that sore throat was associated with co-occurring mutations in HA and NA of A(H1N1)pdm09.

**Table 2:** Demographic and clinical characteristics of patients in different groups

	Patients with co-occurring mutations in HA and NA (n = 10)	Patients without co-occurring mutations in HA and NA (n = 10)	*P*
Male sex (%)	4 (40.0)	4 (40.0)	> 0.05
Age (years)	37.5 (16–63)	48 (27–77)	> 0.05
Coexisting disease (%)	3 (30.0)	4 (40.0)	> 0.05
Fever hours	30.0 ± 17.2	37.2 ± 24.3	> 0.05
Max temperature (°C)	39.1 ± 0.5	39.1 ± 0.4	> 0.05
Headache (%)	5 (50.0)	6 (60.0)	> 0.05
Joint and muscular soreness (%)	7 (70.0)	9 (90.0)	> 0.05
Nasal congestion and rhinorrhea (%)	5 (50.0)	6 (60.0)	> 0.05
Sore throat (%)	10 (100.0)	5 (50.0)	< 0.05
Cough (%)	7 (70.0)	10 (100.0)	> 0.05
Expectoration (%)	3 (30.0)	7 (70.0)	> 0.05
Chestpain (%)	2 (20.0)	0 (0.0)	> 0.05
White blood cell counts (× 10^9^/L)	7.6 ± 3.2	6.5 ± 1.2	> 0.05
Neutrophil (%)	71.5 ± 10.7	73.0 ± 8.2	> 0.05
Neutrophil (× 10^9^/L)	5.5 ± 2.6	4.7 ± 1.2	> 0.05
Lymphocyte (%)	17.0 ± 6.3	15.4 ± 5.6	> 0.05
Lymphocyte (× 10^9^/L)	1.2 ± 0.6	1.0 ± 0.4	> 0.05
Monocyte (%)	10.3 ± 5.0	10.9 ± 3.2	> 0.05
Monocyte (× 10^9^/L)	0.8 ± 0.6	0.7 ± 0.2	> 0.05
Hemoglobin(g/L)	136.1 ± 22.9	139.7 ± 12.7	> 0.05
Platelets (× 10^9^/L)	209.5 ± 53.0	205.3 ± 34.7	> 0.05
C-reactive protein (mg/L)	15.7 ± 9.9	21.2 ± 33.2	> 0.05
Pneumonia (%)	0 (0.0)	1 (10.0)	> 0.05

## Discussion

Since its emergence in 2009, influenza A(H1N1)pdm09 strain has been one of the seasonal influenza strains and circulated seasonally in humans. Genetic evolution of seasonal influenza viruses is gradual. The close monitoring on the viruses are of note for the vaccine strain recommendations. Since 2009, persistent monitoring of the influenza viruses has been conducted in PKUPH in Beijing, China. We have previously studied the evolution of surface antigen site mutations using the viruses circulated in 2012–2014 [19, 20]. During the 2015–2017 influenza season, influenza A viruses have impacted greatly on the public health (Fig. 1). In order to continuously exploring molecular epidemiological evolution, we analyzed the evolutionary and molecular characteristics of the 33 strain A(H1N1)pdm09 virus strains during the 2015–2017. In consistent with the previous study [5], the influenza A(H1N1)pdm09 viruses circulated in 2015–2017 in Beijing, China, were belonged to subclade 6B.1A (Fig. 3). The two genetic subclades may be caused by several amino acid substitutions.

Comparing to the HA protein of A/California/07/2009 vaccine strain, A(H1N1)pdm09 viruses circulated in Beijing during 2012–2014 contained several substitutions in the key epitope (Sa, Sb, Ca and Cb) [19, 20], including H155Q in Ca epitopes, L178I/ K180Q in Sa epitope, and S202T in Sb epitope and so on. In this study, all A(H1N1)pdm09 detected during 2015–2017 had S220T, K180Q, S202T mutations, and no H155Q/R, L178I, G187E mutations was detected. The newly discovered S86T was near Cb epitope. The V169T/S181T was near Sa epitope, and G254 was near Ca epitope. They may affect the antigenic properties of the viruses. Substitutions which affect the viral receptor binding profiles have not been detected. It indicated that the evolutions of A(H1N1)pdm09 viruses were gradually in progress. The emergence of unreported mutations, especially those with potential to affect the viral antigen should be of note monitored.

Correspondingly, substitutions in NA antigen epitope have been reported to occur in the A(H1N1)pdm09 viruses during 2012–2014, including NA- K84N, P93S, V106I L139V, I163T, V264I, E287K and S340F. while in this study, NA-V264I mutation occurred in all the A(H1N1)pdm09 viruses detected during 2015–2017. No mutations of NA-K84N, V106I, L139V, I163T, E287K, or S340F has been detected. Moreover, seven locus mutations were newly found, including A86T (1strain), F115L (4 strains), N449T (4 strains), S450V (4 strains), D451T (4 strains), T452L (4 strains), V453W (4 strains).

Comparing with substitutions in the A(H1N1)pdm09 viruses in 2012–2014, several substitutions have not be detected in 2015–2017. It suggested that these variation sites may only be transient variation. On contrast, the increased prevalence of HA-K180Q and NA-V264I mutations from 2012–2014 to 2015–2017 in all strains suggested that several mutations may gradually become dominant over time under the pressure of human immune selection [21, 22].

Many events such as recombination and infection of new hosts can disrupt the balance between HA and NA, but mutations in the HA and NA genes can make up for this imbalance [23–25]. Co-occurring mutations, including HA-V169T, A278S, E508G, D518E and NA-V67I were found in the new sites compared with vaccine strains A/California/07/2009 and A/Michigan/45/2015 (H1N1). Symptomatically, sore throat was associated with co-occurring mutations in HA and NA of A(H1N1)pdm09.

While our study highlighted the importance of investigating the effect and mechanism of co-occurring mutation of HA and NA; however, this study had several limitations. First, the study population were all from outpatients who were not severely ill, and the findings may not be generalized to hospitalized or severely ill individuals. Second, the medical data collection was limited, and many important clinical data such as further examination, period of treatment, and prognosis were not available. Third, the number of study patients may not be sufficient to draw the firm conclusions. Fourth, co-occurring mutations we observed had possibility of gradual accumulation of mutations, and we analyzed the co-occurring mutation of HA and NA through the clinical manifestations without mechanism. Therefore, further studies with a larger sample size and more clinical data will be needed to confirm and extend our findings. Evidences of increased fitness or epistasis between the HA and NA segments will also be needed in order to exclude gradual accumulation of mutations.

## Conclusions

In this study, we analyzed molecular evolution and amino acid variation characteristics of HA and NA of A(H1N1) pdm09 during the 2015–2017 influenza seasons in Beijing, founding these viruses shared common evolutionary lineages to the vaccine strain A/Michigan/45/2015 (H1N1). However, through our team’s monitoring on molecular evolution and amino acid variation of A(H1N1)pdm09 these years, some variation sites were only transient variation, while some from one to all strains, suggesting that persistent monitoring was needed. Co-occurring mutations of HA-V169T, A278S, E508G, D518E and NA-V67I were detected in 30.3% (10/33) A(H1N1)pdm09 virus strains when comparing with the two vaccine strains A/California/07/2009 and A/Michigan/45/2015 (H1N1). We also made progress on exploring the clinical characteristics of co-occurring mutations in HA and NA proteins of influenza A(H1N1)pdm09 virus. Above results highlight the fact that it is necessary to study the effect and mechanism of co-occurring mutations in the surface antigens HA and NA on patients’ clinical features, and one day those co-occurring mutations maybe used as biomarkers of clinical features to assist the clinical work.

## Supplementary information

**Additional file 1: Table 1.** Nucleotide similarity of HA genes compared to vaccine strains.

**Additional file 2: Table 2.** Amino acid similarity of HA genes compared to vaccine strains.

**Additional file 3: Table 3.** Nucleotide similarity of NA genes compared to vaccine strains.

**Additional file 4: Table 4.** Amino acid similarity of NA genes compared to vaccine strains.

## Data Availability

The datasets used and/or analyzed in the current study are available from the corresponding author upon reasonable request.

## Abbreviations

HA: Hemagglutinin
NA: Neuraminidase
PKUPH: Peking University People’s Hospital
RT-PCR: Reverse transcriptase polymerase chain reaction
NCBI: National Center for Biotechnology Information
CDC: Center for Disease Control and Prevention
